# The knowledge, attitudes and perceptions of physiotherapists in KwaZulu-Natal, South Africa, towards mental health

**DOI:** 10.4102/sajp.v76i1.1483

**Published:** 2020-10-30

**Authors:** Marilyn Hooblaul, Saul Cobbing, Kurt J. Daniels

**Affiliations:** 1 KwaZulu-Natal Department of Health, Ekuhlengeni Psychiatric Hospital, Durban, South Africa; 2 Department of Physiotherapy, University of KwaZulu-Natal, Durban, South Africa

**Keywords:** physiotherapy, mental health, attitudes, knowledge, perceptions

## Abstract

**Background:**

Physiotherapists are trained to manage patients with physical needs, but because of limited training at an undergraduate level in mental health, patients may not receive holistic care. This lack of knowledge often can deny people living with a mental illness (PLWMI) the potential benefits of physiotherapy treatment and exercise.

**Objectives:**

Our study conducted in the KwaZulu-Natal province of South Africa aimed to determine the knowledge, attitudes and perceptions of physiotherapists working in the Department of Health (DoH) in KwaZulu-Natal, South Africa, towards mental health, and to determine whether their undergraduate training prepared them to manage PLWMI.

**Methods:**

A mixed-method design was employed. The Attitudes to Psychiatry (ATP-30) questionnaire was distributed to 153 physiotherapists in KwaZulu-Natal. Focus groups were conducted to ascertain whether their undergraduate training prepared them adequately to manage PLWMI.

**Results:**

A total of 124 physiotherapists completed the questionnaires. The mean ATP-30 scores was 103.70 (SD = 11.71). Females had slightly higher ATP-30 scores than males. Physiotherapists indicated in the focus groups that they received limited training about mental health at an undergraduate level.

**Conclusion:**

Physiotherapists working in the KwaZulu-Natal public sector have a positive attitude towards mental health and managing PLWMI. Participants expressed the need for the inclusion of theoretical and practical knowledge about mental health in the undergraduate curriculum and postgraduate courses related to this topic.

**Clinical implications:**

The outcomes of this study show the importance of the inclusion of mental health in the undergraduate physiotherapy programme. Exposure to the theoretical and practical knowledge of mental health during the undergraduate physiotherapy programme will assist qualified physiotherapists better manage PLWMI. Future studies should be conducted in the other provinces and in the private practice setting in South Africa, so as to compare the results.

## Introduction

There are more than 45 million people living with a mental illness (PLWMI) globally. Almost one-third of the population of South Africa suffers from mental or neurological disorders, thus placing mental illness among the leading causes of ill-health and disability (Vancampfort et al. 2018). Kolappa, Henderson and Kishore (2013) have stated that there can be ‘No health without mental health’. However, nearly, 75% of PLWMI never seek medical treatment because of stigmatisation, discrimination and neglect (World Health Organization 2013). The prevalence of stigmatisation and discrimination towards PLWMI has been shown in extensive studies (Probst & Skjaerven 2017) and arises from fear, lack of knowledge, apathy and negative stereotypes (Egbe et al. 2014). Because of stigmatisation, PLWMI are reluctant to access medical treatment (Ay, Save & Fidanoglu 2006) as stigmatisation is not only prevalent in the community, but health care professionals are also guilty of stigmatising PLWMI (Egbe et al. 2014; Sinawi & Alawi 2016). This can be reduced by knowledge, compassion and understanding (Pauw 2011). Yildirim et al. (2015) state that the undergraduate physiotherapy programme should include mental health to prevent stigmatisation and promote positive attitudes to manage PLWMI without prejudice (Connaughton & Gibson 2016a, 2016b; Dandridge et al. 2014; Probst & Peuskens 2010; Yildirim et al. 2015). Connaughton and Gibson (2016a) have shown that education can foster positive attitudes. Physiotherapists with limited knowledge about mental health can use their personal experiences, negative stereotypes and prejudices to determine their management of PLWMI (Connaughton & Gibson 2016a).

Extensive research has been conducted about the link between physical and mental health, although many health care professionals are unable to manage PLWMI holistically (Connaughton & Gibson 2016a). According to the International Organization of Physical Therapists in Mental Health (IOPTMH), which is a branch of the World Confederation of Physical Therapy, physiotherapy in mental health aims to assist PLWMI by promoting functional movement, physical activity and exercises linking physical and mental issues based on scientific and clinical evidence (World Confederation of Physical Therapy 2016). Physiotherapy plays an integral role in the management of patients with musculoskeletal conditions and could therefore also play an important role in the treatment of mental health disorders. Demyttenaere et al. (2007) show that musculoskeletal conditions such as chronic neck or back pain are associated with mental health disorders. Connaughton and Gibson (2016b), however, reveal that physiotherapy students are unaware of the prevalence of co-morbidities in PLWMI, and they are also intrigued to learn about the relationship between chronic pain and mental health.

Physiotherapy management includes the prescription of physical activity for patients. There is evidence that shows that physical activity enhances the effectiveness of psychological therapies (Probst & Skjaerven 2017) and quality of life of PLWMI (Richardson et al. 2005). The most common form of physical activity is exercise. The benefits of exercise for PLWMI are improved mood, sleep patterns, cognitive function, self-esteem, quality of life (Kaur & Garnawat 2009), relief from stress, increased energy and reduction in cholesterol levels, and these benefits can help with weight reduction (Kaur, Masaun & Bhatia 2013). Therefore, physiotherapists can play an important role in improving mental as well as the physical health of patients (Kaur et al. 2013). Numerous studies highlight the close relationship between mental and physical health (Probst & Skjaerven 2017), but integrating this into clinical settings has been very slow, and physiotherapy has not been seen as a worthwhile strategy (Probst & Skjaerven 2017). A study conducted in Nigeria reports that there is a need for the integration of physiotherapy management into mental health (Gbiri, Akinpelu & Odole 2011), and physiotherapists are seen as an important member of the mental health team (Gbiri et al. 2011).

In South Africa, eight universities offer physiotherapy undergraduate programmes and the curricula reflect that the focus of teaching is primarily on the physical aspect of health with limited focus on mental health. There is no evidence, however, on the knowledge, attitudes and perceptions of physiotherapists in KwaZulu-Natal province towards mental health and their perceived preparedness to manage PLWMI. Our study aimed to determine the self-reported knowledge, attitudes and perceptions of qualified physiotherapists working in the public sector in KwaZulu-Natal, South Africa, towards mental health and to determine whether their undergraduate training prepared them to manage PLWMI.

## Method

A mixed-method study using an online questionnaire and focus group interviews was conducted in KwaZulu-Natal, South Africa. The questionnaire included the demographic details of the participants, the university where they completed their undergraduate physiotherapy studies, how frequently they treated PLWMI and questions from the Attitudes to Psychiatry (ATP-30) questionnaire (Burra, Kalin & Leichner 1982). The ATP-30 tool has been used in three studies measuring the attitudes of physiotherapy students and one study measuring the attitudes of physiotherapists, and therefore it was possible to compare the results to other studies by using the same questionnaire (Bhise et al. 2016; Gbiri et al. 2011; Probst & Skjaerven 2017; Sinawi & Alawi 2016). The tool has been shown to be reliable and valid with medical and occupational therapy students (Burra et al. 1982).

Participants in our study were qualified physiotherapists employed by the Department of Health (DoH) in KwaZulu-Natal, South Africa. A total population sampling method was employed (McCusker & Gunaydin 2015).

### Data collection

All managers of DoH physiotherapy departments at KwaZulu-Natal hospitals were sent a link to the online questionnaire via email. The email provided detailed information on our study and included a consent form for participants to sign. The managers were requested to inform all the physiotherapy staff (*n* = 153). The study information sheet, consent form and questionnaires were hand-delivered and collected from the physiotherapists at facilities. Access was granted to conduct our study within the eThekwini District of KwaZulu-Natal. The first author ensured that those participants who had received hand-delivered copies only filled in the questionnaire once. Purposive sampling was used to recruit physiotherapists for the three focus groups, based on locality and demographics (Tongco 2007). Questions posed to focus group participants were open-ended and were not in the questionnaires. Three focus groups were conducted with a total of 24 participants. The two open-ended questions participants were asked to focus the discussion on were:

- What knowledge have you acquired since graduation about mental health?
- What would you have liked to know about mental health before graduating?

The reason for posing the first question was to ascertain if participants had gained any knowledge about mental health through their years of experience and how they had done so. The second question was to ascertain as to whether they felt prepared after undergraduate physiotherapy training to manage PLWMI. All the information gained in the focus groups was recorded by using an audio-recorder.

### Data analysis

Completed questionnaires received were captured on an Excel spreadsheet. The data obtained from the questionnaires were numerically analysed by determining the mean, mode, range, variance and standard deviation. The quantitative data were analysed by using an SPSS programme. The data are graphically presented in tables. The results from the ATP-30 are presented as means and standard deviation, and the results compared with similar studies.

Linear regression outputs (α = 0.05) were used to compare gender, frequency of treating PLWMI and personal interaction with PLWMI. The data from the questionnaire were compared with studies using the same ATP-30 tool. The data captured from the focus groups were analysed by using a qualitative content analysis process which is to identify common themes or concepts (Stemler 2001). The Nvivo programme was used to analyse the qualitative data. Emergent themes were identified from the qualitative data. The first author used the grouped responses to group subthemes by using repetition of responses to the two questions posed to participants. Content analysis has been used extensively in psychiatry as it has many benefits (Elo & Kynga 2007). Deductive content analysis was employed to allow comparisons to a previous study (Connaughton & Gibson 2016a).

### Ethical consideration

Ethical approval was granted by the University of KwaZulu-Natal Biomedical Research Ethics Committee (ethical clearance number BE389/19). Gatekeeper permission was sought from the KwaZulu-Natal Department of Health (KZ_201906_032). Every participant provided written consent, they were informed about the purpose of the study and that their participation was voluntary, and they could withdraw from our study at any time. Participants were informed that all data collected would remain confidential. Anonymity, confidentiality and privacy of rights were maintained throughout our study.

## Results

A total of 125 participants completed the questionnaire. One individual did not complete all the questions and therefore was not included in the analysis. The final number of participants considered for analysis was thus 124, representing 81% of the total population. Participants’ ages ranged between 26 and 57, with a mean age of 38 (SD 7.5). There were 106 (85.5%) female participants.

Participants who responded had a positive attitude towards mental health with a mean ATP-30 score of 103.70 (SD 11.71). Female and male participants had the same ATP-30 scores. Participants’ grouped characteristics relative to the ATP-30 scores are presented in Table 1.

**TABLE 1:** Selected participant characteristics and their influence on the Attitudes to Psychiatry-30 scores by using a linear regression model.

Characteristic	Total		ATP scores		*p*
	*n*	%	*n*	SD	
**Gender**					
Female	106	85.5	103.84	11.39	-
Male	18	14.5	102.82	13.88	0.292
**Participants’ number of years of experience as a physiotherapist**					
0–5 years	4	3.2	94.75	4.57	-
6–10 years	40	32.3	102.45	11.62	0.813
+ 10 years	80	64.5	104.78	11.83	0.924
**Participants who have never treated PLWMI**	19	15.4	102.21	12.02	-
**Participants who have treated PLWMI**	105	84.6	103.97	11.68	0.167
**Participants who have friend or family member with a mental illness**	84	67.7	104.10	12.05	0.894
**Participants / do not have friend or family member with a mental illness**	40	32.3	102.88	11.05	-
**Frequency of treating PLWMI**					
Never	19	15.3	102.47	12.30	-
Almost every day	2	1.6	123.53	37.80	0.015†
3–4 times a week	5	4.0	97.40	3.21	0.322
1–2 times a week	21	16.9	106.29	11.84	0.183
Twice a month	23	18.5	102.40	10.32	0.172
Once a month	54	43.5	105.00	11.53	0.151

ATP, attitudes to psychiatry; SD, standard deviation; PLWMI, people living with a mental illness.

† , Indicates the corresponding variable significantly affects ATP scores at α = 5%.

Female participants were shown to have treated PLWMI more frequently than male participants. Participants with a family member or friend with a mental illness had treated PLWMI more frequently than participants who did not have a family member or friend with a mental illness. Selected characteristics of participants relative to the frequency of treatment of PLWMI are presented in Table 2.

**TABLE 2:** The influence of frequency of treatment of people living with a mental illness on selected participant characteristics by using a binary logistic regression model.

Variables	Frequency of treatment of PLWMI				Adjusted odds ratio	95% CI	*p*
	Never treated PLWMI		Have treated PLWMI				
	*n*	%	*n*	%			
**Gender**							
Female	11	10.4	95	89.6	3.99	1.94–12.93	0.004
Male (ref)	8	44.4	10	55.6	1.00	-	-
**Years of experience**							
0–5 years	1	25.0	3	75.0	7.42	0.46–11.80	0.157
6–10 years	3	7.5	37	92.5	2.73	0.65–11.43	0.169
> 10 years (ref)	15	18.8	65	81.3	1.00	-	-
**Family**							
No	14	35.0	26	65.0	0.13	0.04–0.43	0.001
Yes (ref)	5	6.0	78	94.0	-	-	-

CI, confidence interval; PLWMI, people living with a mental illness.

Key themes that emerged from the focus groups are represented in Table 3. Participants expressed that directly after graduation they were not prepared to manage PLWMI because of the limited knowledge about mental health they received at an undergraduate level. Participants stated that their knowledge after graduation was gained through working with PLWMI and from other members of the multidisciplinary team. Participants also expressed that they would have liked to have known more about the effects of mental health on physical health.

**TABLE 3:** Emergent themes from focus groups.

Themes	Subthemes	Illustrative quotes
Knowledge of physiotherapists acquired since graduation about mental health	**Prevalence of mental illness and stigma** Participants expressed concerns about the prevalence of mental illness. They have noted treating more PLWMI than in previous years. They also stated that they are also guilty of stigmatising PLWMI because they have had no exposure of treating PLWMI at the undergraduate level; they rely on their own perceptions and negative stereotypes.	‘The training is required more now than in previous years because of the demands on social life and the threats to society, and stressors placed on life are on the increase. There should be a greater awareness amongst physiotherapists about mental health’. (Participant 4, focus group 2)
		‘No exposure at the undergraduate level will lead to negative perceptions and fear of PLWMI. Mental illness has always been there, but we shy away from conditions we don’t understand’. (Participant 5, focus group 3)
		‘When the referral comes from the psychiatric ward for physiotherapy, there is no one that will volunteer to go, as we are afraid of past experiences of other physiotherapists reporting aggressive patients. We don’t know what to expect from the patients and we fear for our safety and the safety of the patient’. (Participant 1, focus group 1)
		‘I don’t want to work in a psychiatric hospital because I am scared, all the patients will be aggressive like I see on TV’. (Participant 4, focus group 1)
	**Self-learning** Participants stated that because of the lack of knowledge at the undergraduate level about mental health, their knowledge gained has been from working with PLWMI over the years, and this was a disadvantage to that patient group. The knowledge that they have gained over the years has been self-taught, and working with other members of the multi-disciplinary team that have knowledge about mental health.	‘The knowledge that I have gained is a working knowledge that has been gained through years of working, I have been using a trial-and-error approach because I lack the proper education about mental health’. (Participant 4, focus group 3)
		‘I have attended in-service training about mental health from other disciplines at my institution’. (Participant 6, focus group 3)
	**The role of physiotherapy in mental health** Some participants were surprised that after graduation they had a role to play in mental health. Participants mentioned that after graduation they solely believed that their role was purely a physical one, and they were only going to see conditions that they were taught at the undergraduate level. Participants were not aware that physiotherapy treatment modalities can be used to treat PLWMI.	‘I had no idea that I had to treat PLWMI after graduation; I didn’t know what my role was as a physiotherapist. I attended a conference and I heard a physiotherapist talking about mental health and I was utterly surprised’. (Participant 1, focus group 3)
		‘Not sure how I can treat a patient just with a mental illness’. (Participant 4, focus group 2)
Knowledge that physiotherapists wish they had acquired before graduation about mental health	**Effects of mental health on physical health** Participants stated that they were not aware that mental illness can affect a patient’s physical health. They solely believed that they were only responsible for managing the physical illness of a patient. To understand how mental illness affects physical health, education is required about signs and symptoms and aetiology about mental health as well and the medication and its side effects.	‘It is difficult to treat a patient with a co-morbid mental illness when we don’t understand the effects of the medication as it affects the rehabilitation of the patients and that leads to us assuming the patient is being difficult or lazy’. (Participant 1, focus group 1 )
		‘I wish we had learnt about the different mental illnesses as well as how they affect the physical health of a patient, then I would be able to manage patients’. (Participant 3, focus group 1)
	**Communication strategies** Participants stated that they lacked proper communication strategies when managing PLWMI and thus making treatment sessions far longer. They expressed their frustrations for not being able to do their job because they were unable to fully relay instructions to PLWMI. The consensus was if they had received the relevant theoretical knowledge followed with the practical exposure at the undergraduate level, they would be able to better manage PLWMI.	‘I should have been equipped with communication strategies at undergraduate training, as it is not easy to manage a patient with dementia; sometimes they don’t understand simple instructions and then it becomes difficult to do your job’. (Participant 4, focus group 2)
		‘I would like to know how to manage a patient with depression or anxiety that has been attributed from the physical condition, like a patient with paraplegia experiencing depression’. (Participant 2, focus group 2)
		‘If a patient tells me they want to kill themselves, I don’t know what to do, I need to have some skills on counselling’. (Participant 5, focus group 1)
	**Referral processes** Participants would have liked to know how the referral process works and what the roles of the other members of the multi-disciplinary team are. They expressed their concerns about not being confident in referring PLWMI to other disciplines.	‘I don’t know how to refer a patient who is undiagnosed with a mental illness. I don’t know when to refer to a psychologist or psychiatrist’. (Participant 4, focus group 1)
		‘I’m not sure if a patient who is undiagnosed requires a social worker or occupational therapist and if those professions manage those patients’. (Participant 6, focus group 2)

PLWMI, people living with a mental illness.

## Discussion

The key findings of our study will be discussed in relation to the study aim, which was to investigate the knowledge, attitudes and perceptions of physiotherapists in KwaZulu-Natal, South Africa, towards mental health and their preparedness to manage PLWMI after graduation. The findings suggest that despite participants reporting a paucity of knowledge regarding PLWMI, they demonstrated a mostly positive attitude towards treating these patients. This paucity of knowledge could contribute to the poor perceptions that many physiotherapists described in the focus group sessions. We found there were more female participants than males. This difference in the number of male and female participants is because of the fact that more females pursue a physiotherapy career path, and the sample is thus representative of the population.

Knowledge can be defined as factual information acquired through learning (Geer et al. 2006). The collected data suggest that participants lacked self-reported knowledge about mental health. They expressed a need for structured undergraduate learning, which includes both theoretical and clinical components to enhance their knowledge of PLWMI. A study conducted at the University of KwaZulu-Natal found that both clinical and theoretical teaching is vital to students’ learning (Chetty et al. 2018), and Probst and Peusken (2010) also deduced a similar finding but added that course work was not sufficient to improve attitudes, but there needs to be direct contact with PLWMI. Countries such as Belgium, Norway and Sweden offer training on mental health at both undergraduate and postgraduate levels. Physiotherapists in our study stated that having more knowledge about aetiology, signs and symptoms of mental illness and side effects of medication would enable them to treat patients holistically. Other studies on physiotherapy students have similar findings and identify the need for more education related to mental health (Connaughton & Gibson 2016b; Dandridge et al. 2014; Probst & Peuskens 2010; Yildirim et al. 2015). Participants in the focus groups also stated they have learnt about mental health through in-service training and years of working with PLWMI; this has not been shown in other studies.

Attitude can be defined as an emotion, feeling or desire for learning (Geer et al. 2006). Our participants have a relatively positive attitude towards mental health, which is similar to the study by Connaughton and Gibson (2016a), which is the only other study to explore the knowledge, attitudes and perception of physiotherapists as well as those studies that have explored attitudes of physiotherapy students towards mental health (Bhise et al. 2016; Connaughton & Gibson 2016b; Dandridge et al. 2014; Probst & Peuskens 2010; Yildirim et al. 2015). There was no significant difference in the ATP-30 scores between male and female participants, which differs from Connaughton and Gibson’s (2016a) study on physiotherapists and physiotherapy students (Bhise et al. 2016; Connaughton & Gibson 2016b; Probst & Peuskens 2010).

Twenty-one per cent of physiotherapists reported treating patients with co-morbid mental illness at least once a week, which is lower than that in the study by Connaughton and Gibson (2016a) who found that 75% of physiotherapists treat patients with co-morbid mental illness at least once a week. The difference in treatment rates could be explained by the non-disclosure of PLWMI to health care professionals because of the fear of being judged and stigmatised (Pauw 2011). In South Africa, 75% of the population with a mental illness will not receive any treatment in their lifetime, with the majority of these individuals coming from middle- and low-income households. Participants who treated PLWMI daily had a more positive attitude than participants who had never treated PLWMI. This more positive attitude could be attributed to the constant interaction with PLWMI. This finding was similar to that of Connaughton and Gibson (2016a), who showed that physiotherapists who had constant interaction with PLWMI had a more positive attitude towards mental health and PLWMI. Yildirim et al. (2015) and Probst and Peuskens (2010) demonstrated that education about mental health could promote a positive attitude in physiotherapy students at an undergraduate level.

Perception can be defined as an interpretation of information based on an individual’s understanding of a subject (Geer et al. 2006). We were able to interpret the perceptions of the participants through the focus groups. They expressed a perception that when treating PLWMI, because of their fear and lack of understanding of mental health, they are forced to rely on what they know. One of the participants stated, ‘I don’t want to work in a psychiatric hospital because I am scared; all the patients will be aggressive like I see on TV’. This stigmatisation can jeopardise the patient–therapist relationship, patient adherence to treatment and management of the patient (Synnott et al. 2015). We found that participants with a family member or friend with a mental illness had treated PLWMI more frequently, which may therefore suggest that the interaction with PLWMI greatly reduces stigmatisation and modifies attitudes towards mental health. To reduce stigmatisation and inculcate a positive attitude, physiotherapy training at an undergraduate level should equip students with knowledge and skills through interaction with PLWMI, to manage PLWMI without prejudice. It was noted from the focus groups that participants were very reluctant to work in psychiatric hospitals, despite the importance of physiotherapeutic interventions in mental health care facilities (Kaur et al. 2013).

Our study also aimed to explore whether qualified physiotherapists working in the public sector in KwaZulu-Natal, South Africa, were adequately prepared through their undergraduate physiotherapy training to manage PLWMI. Responses from the focus groups highlighted that participants felt that they were underprepared to manage PLWMI effectively. A similar finding was noted in an Australian study (Probst & Skjaerven 2017). Participants stated that they were ill-equipped with communication skills and knowledge when managing PLWMI after graduation and therefore had to learn about the aetiology of mental illness and learn to communicate through their interactions with PLWMI. This knowledge was gained through years of experience. There was also a concern for the future generation of physiotherapists, particularly as studies indicate that the prevalence of mental illness is increasing in South Africa (Herman et al. 2009). Although the participants reported that they had insufficient knowledge about mental health, the overall attitudes towards mental health were positive. So their knowledge did not seem to affect their attitudes towards mental health, but it did affect their perceptions of managing PLWMI.

## Strengths and limitations

To our knowledge, this is the first study conducted on the knowledge, attitudes and perceptions of qualified physiotherapists towards mental health in South Africa. There was a very good response rate to our study, with 124 out of a possible 153 (81%) completing the questionnaire. This compares favourably to previous studies using email questionnaires (Sheehan 2006). A limitation of our study may have been the use of the ATP-30 tool, as it was designed for medical doctors and occupational therapists. There is currently no physiotherapy-specific tool to measure attitudes of physiotherapists towards mental health. The undergraduate curricula were also not interrogated at the universities that offer physiotherapy training. A review of the curricula would provide insight into the mental health content. Physiotherapists were not asked if they attended post-graduate or continuous professional development training on mental health.

## Recommendations

Further studies should be conducted in the other provinces of South Africa, as well as with physiotherapists working in private practice to compare our results with studies from other settings. Our results indicate the need for the undergraduate physiotherapy programme to be reviewed to include mental health studies. Undergraduate physiotherapy students need to acquire knowledge about aetiology, signs and symptoms about mental illness, medications and side effects, and communication strategies on managing PLWMI. Physiotherapy students need to implement their theoretical teaching in a clinical setting as well, and this requires an increased exposure to managing PLWMI. Post-graduate courses about mental health will assist physiotherapists to ensure that they are equipped with the knowledge to manage PLWMI effectively.

## Conclusion

Our study found that physiotherapists working in public sector hospitals in KwaZulu-Natal province, South Africa, had limited self-reported knowledge about mental health after graduation. Despite the challenges resulting from their limited undergraduate programme, physiotherapists have a positive attitude towards mental health. They were able to gain knowledge by themselves or by attending in-service training by members of the multidisciplinary teams with expertise on mental health. As there is an alarming annual increase in the number of patients in South Africa presenting with mental health problems, physiotherapists will have increased interactions with PLWMI whilst in practice and need to be equipped with skills and knowledge to manage these patients. There is thus a need for a review of the undergraduate physiotherapy curricula in South Africa to enhance the inclusion of mental health content in all curricula as well as to increase the number of postgraduate continual professional development activities related to physiotherapy in the management of PLWMI.
